# Common clonal origin of three distinct hematopoietic neoplasms in a single patient: B-cell lymphoma, T-cell lymphoma, and polycythemia vera

**DOI:** 10.1101/mcs.a006313

**Published:** 2023-12

**Authors:** Dingani Nkosi, Andrew W. Allbee, Paul G. Rothberg, Jonathan W. Friedberg, Andrew G. Evans

**Affiliations:** 1 Department of Pathology and Laboratory Medicine, University of Rochester, Rochester, New York 14642, USA;; 2 University of Rochester School of Medicine and Dentistry, University of Rochester, Rochester, New York 14642, USA;; 3 Wilmot Cancer Institute, University of Rochester, Rochester, New York 14642, USA

**Keywords:** B-cell lymphoma, hematological neoplasm, increased hemoglobin, increased red blood cell mass, T-cell lymphoma/leukemia

## Abstract

The potential for more than one distinct hematolymphoid neoplasm to arise from a common mutated stem or precursor cell has been proposed based on findings in primary human malignancies. Particularly, angioimmunoblastic T-cell lymphoma (AITL), which shares a somatic mutation profile in common with other hematopoietic malignancies, has been reported to occur alongside myeloid neoplasms or clonal B-cell proliferations, with identical mutations occurring in more than one cell lineage. Here we report such a case of an elderly woman who was diagnosed over a period of 8 years with diffuse large B-cell lymphoma, polycythemia vera, and AITL, each harboring identical somatic mutations in multiple genes. Overall, at least five identical nucleotide mutations were shared across multiple specimens, with two identical mutations co-occurring at variable variant allele frequencies in all three specimen types. These findings lend credence to the theory that a common mutated stem cell could give rise to multiple neoplasms through parallel hematopoietic differentiation pathways.

## INTRODUCTION

Cancer stem cell theory predicts that multiple terminally differentiated malignancies may arise from a single clonal progenitor stem cell. In rare clinical cases, the potential exists to find and document phenotypically distinct tumor types that arise from a common clonal progenitor. Hematopoietic malignancies, in particular, provide an opportunity to study discrete cancers occurring at different times and in disparate locations and evaluate clonality by means of next-generation sequencing (NGS) for shared somatic mutations.

We present a case of a 79-yr-old woman who was initially diagnosed with low-grade B-cell non-Hodgkin lymphoma transformed to diffuse large B-cell lymphoma (DLBCL), followed by polycythemia vera (PV), and then angioimmunoblastic T-cell lymphoma (AITL) within a period of 8 years. Multiple somatic mutations, each occurring at variable variant allele frequencies (VAFs), were identified across all three different specimen types with at least one “founder” genomic alteration, *TET2* H1382R, detected in all three specimen types. Our case reinforces previous publications showing multilineage development of hematolymphoid malignancies from a common mutated stem cell precursor.

## CASE PRESENTATION

**Figure 1.**
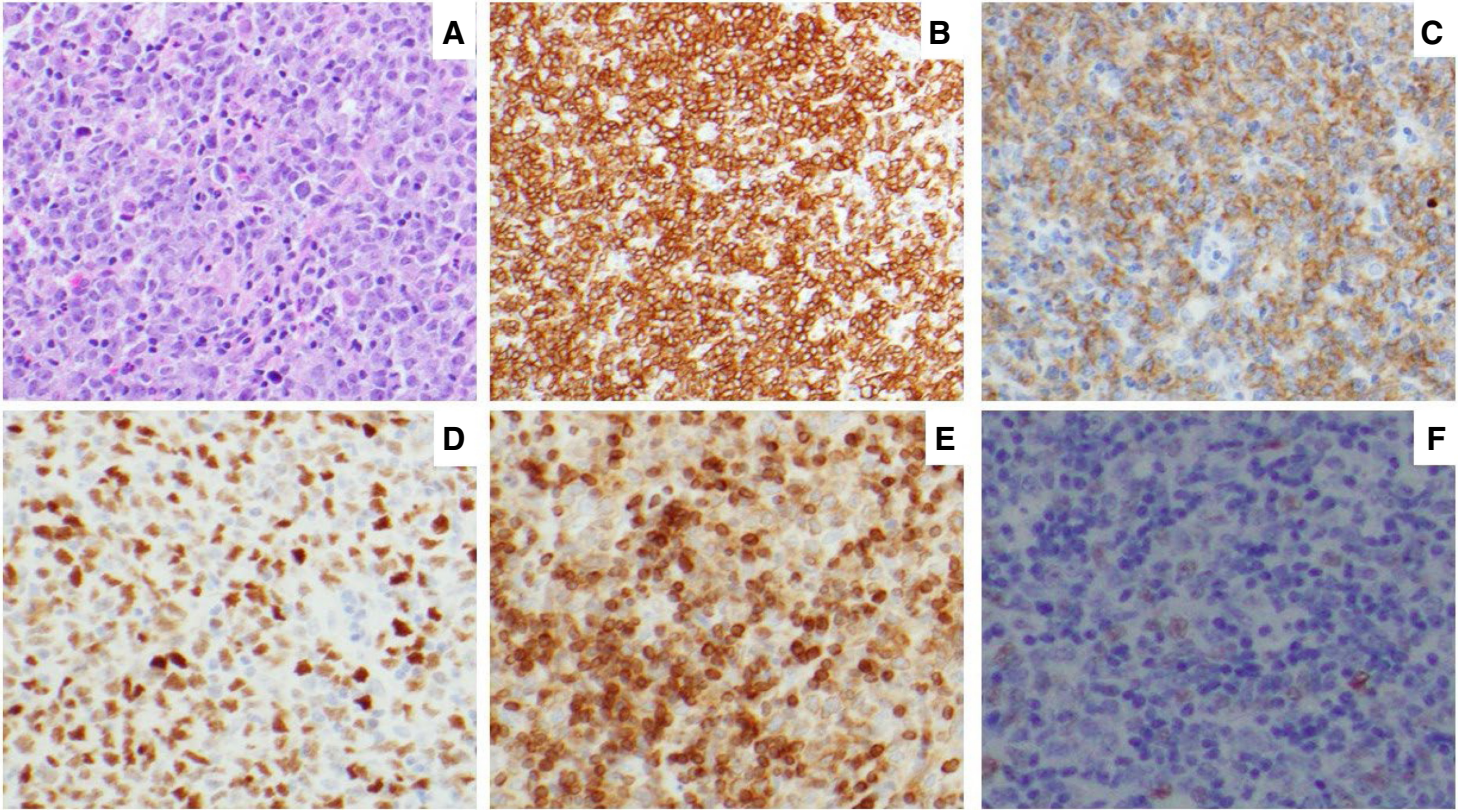
Cavernous sinus mass histology and corresponding immunohistochemical stains. (*A*) Hematoxylin and eosin (H&E) showed diffuse large lymphocytes. (*B–F*) Immunohistochemistry for CD20 (*B*), CD10 (*C*), BCL6 (*D*), BCL2 (*E*), and MYC (*F*).

A 79-yr-old woman developed a sudden onset of right vision loss and right eye ophthalmoplegia. Her past medical history included extranodal marginal zone lymphoma of mucosa-associated lymphoid tissue, diagnosed on small bowel resection 2 years earlier due to obstruction. Magnetic resonance imaging of the head showed a 3-cm mass in the right cavernous sinus extending into the sphenoid sinus. No other hypermetabolic lymphadenopathy or abnormal uptake was visualized within the liver, spleen, or bone marrow by positron-emission tomography (PET) imaging. A transsphenoidal biopsy of the mass showed diffuse large lymphocytes with vesicular chromatin, prominent nucleoli, and irregular nuclear borders (Fig. 1A). Flow cytometry showed clonal B cells positive for CD20, CD10, and κ restricted. Immunohistochemical analysis showed that the tumor cells were positive for CD20 (Fig. 1B), CD10 (Fig. 1C), BCL6 (Fig. 1D), and BCL2 (Fig. 1E) but negative for MYC (Fig. 1F). No abnormalities were seen among intratumoral T cells by CD3 or CD5 immunohistochemistry. The ki-67 proliferation index was 70%. Staging bone marrow biopsy showed normocellular marrow with trilineage hematopoiesis. Cerebrospinal fluid analysis was negative. She was diagnosed with DLBCL without CNS involvement, for which she received six cycles of immunochemotherapy (R-CHOP) and four cycles of intrathecal methotrexate, achieving complete remission.

Five years later, she presented with episodes of leukocytosis (white blood cell count = 20 × 10^3^ cells/µL, with absolute neutrophil count = 17.4 × 10^3^ cells/µL), thrombocytosis (554 × 10^3^ platelets/µL), and erythrocytosis (Hct = 55%) over several months without any B symptoms. She was treated with oral antibiotics for a presumed urinary infection but the cell counts did not normalize. Imaging studies did not show any evidence of lymphadenopathy or recurring DLBCL. Further workup showed positive *JAK2* V617F mutation via conventional allele-specific PCR, with negative FISH study for BCR::ABL1 rearrangement. She was clinically diagnosed with PV and was initially managed with intermittent phlebotomy at least once per month but continued to have elevated hematocrit (∼50%) and fatigue. Low-dose hydroxyurea (500 mg/d) was added to her treatment and later increased to 1000 mg/d for persistent polycythemia. Because of side effects associated with increased dosing, she was returned to low-dose hydroxyurea and intermittent phlebotomy as needed.

**Figure 2.**
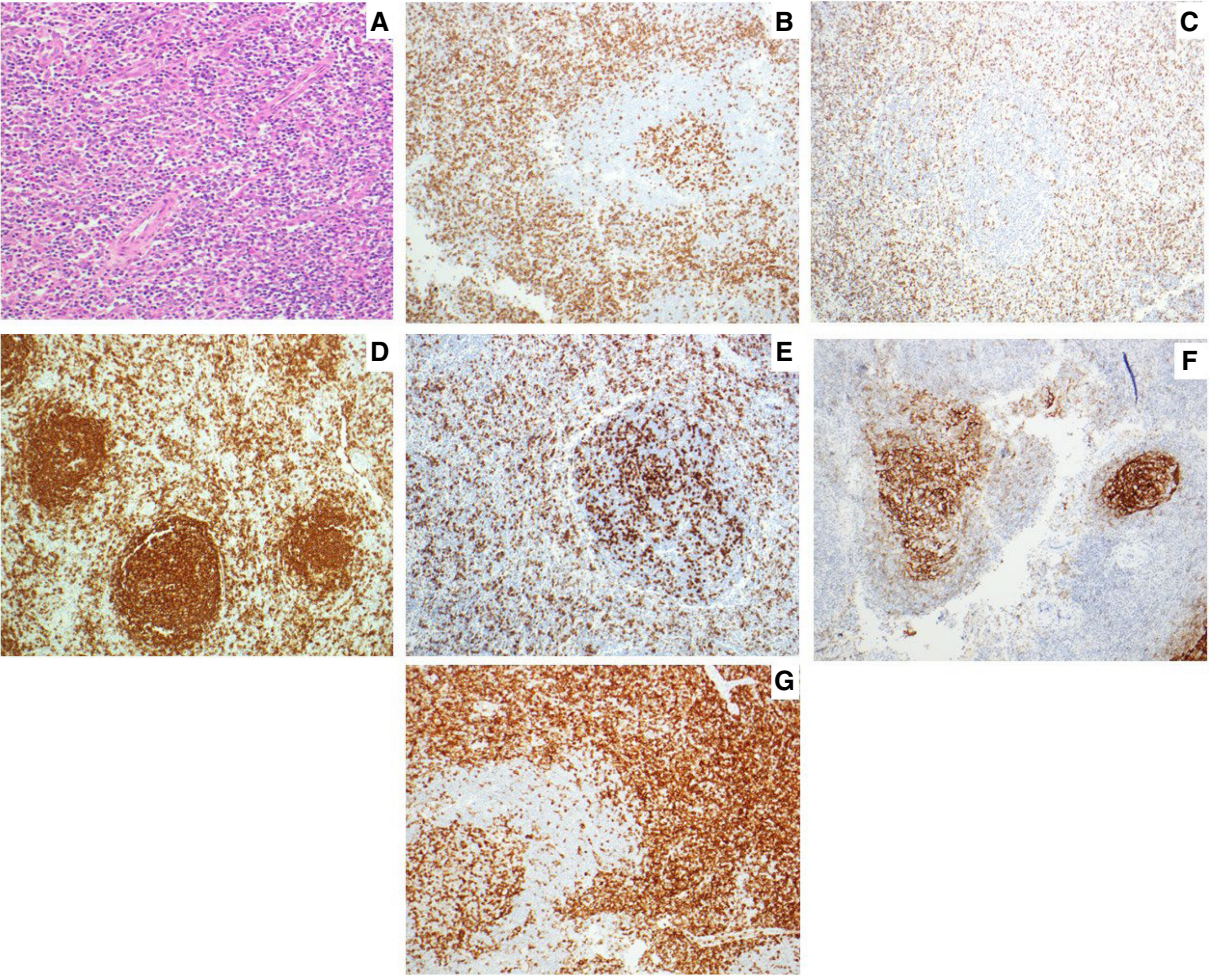
Lymph node biopsy and corresponding immunohistochemical stains. (*A*) Hemotoxylin and eosin (H&E) showing medium-sized lymphocytes with arborizing high endothelial venules. (*B–G*) Immunohistochemistry for CD2 (*B*), CD3 (*C*), CD20 (*D*), PD1 (*E*), CD23 (*F*), and CD4 (*G*).

Seven years after the DLBCL diagnosis, she presented with an enlarged left cervical lymph node. Furthermore, no evidence of lymphadenopathy was seen at other sites on imaging, and the right sphenoid sinus showed features suggestive of chronic sinusitis. Excisional biopsy of the left level II lymph node was performed and showed partial effacement of the lymph node with expansion of interfollicular spaces by small to medium-sized lymphocytes and infiltrated germinal centers. The biopsy also showed some sections with arborizing high endothelial venules (Fig. 2A). The lymphocytes of interest present in interfollicular spaces were positive for CD2 (Fig. 2B), CD3 (Fig. 2C), PD1 (Fig. 2E), and CD4 (Fig. 2G), with expanded and disrupted follicular dendritic cell meshworks expressing CD23 (Fig. 2F). CD20 stained residual follicular and few interfollicular B cells (Fig. 2D). CD30 weakly highlighted rare immunoblasts, negative for CD15, EBER ISH, and ALK1. Flow cytometric analysis revealed polytypic B cells (κ:λ ratio of 1.5), with T cells (54% of lymphocytes) showing a markedly skewed CD4:CD8 ratio (10) but no definitive aberrant loss of pan T-cell antigens tested (CD2, CD5, CD7, or TCRα/β).

Molecular testing was positive for monoclonal rearrangement of the T-cell receptor γ chain (*TCRg*) gene, whereas it was negative for clonal immunoglobulin heavy chain (*IGH*) or κ light chain (*IGK*) gene rearrangements. Somatic mutational analysis for *RHOA* and *STAT3* were both negative. NGS analysis with a clinically validated 34-gene panel (Illumina) was then performed, revealing five different somatic mutations involving three genes: *TET2*, *JAK2*, and *CBL* (Table 1; Results below). The histologic, immunophenotypic, and genetic features were most consistent with AITL. The patient initially had stable disease after receiving seven cycles of brentuximab, but it was later stopped because of severe neuropathy. The patient died 1 yr after discontinuing therapy due to disease progression and associated comorbidities.

**Table 1.** Mutations identified from the different tumor tissues using the two different NGS systems (Illumina and the ArcherDx) with their corresponding VAFs

Gene	Mutation	VAFs			
		DLBCL 2010 (Archer)	Blood 2016 (Illumina)	AITL 2018 (Illumina)	AITL 2018 (Archer)
*JAK2*	V617F (c.1849G > T)	ND	80%	15%	15%
*TET2*	Y1255Ter (c.3765delC)	ND	50%	12%	13%
*TET2*	C1298fsTer65 (c.3893delG)	36%	ND	18%	21%
*TET2*	H1382R (c.4145A > G)	35%	53%	37%	37%
*CBL*	R420Q (c.1259G > A)	10%	2%	26%	25%

(NGS) Next-generation sequencing, (VAFs) variant allele frequencies, (DLBCL) diffuse large B-cell lymphoma, (ND) not determined.

**Figure 3.**
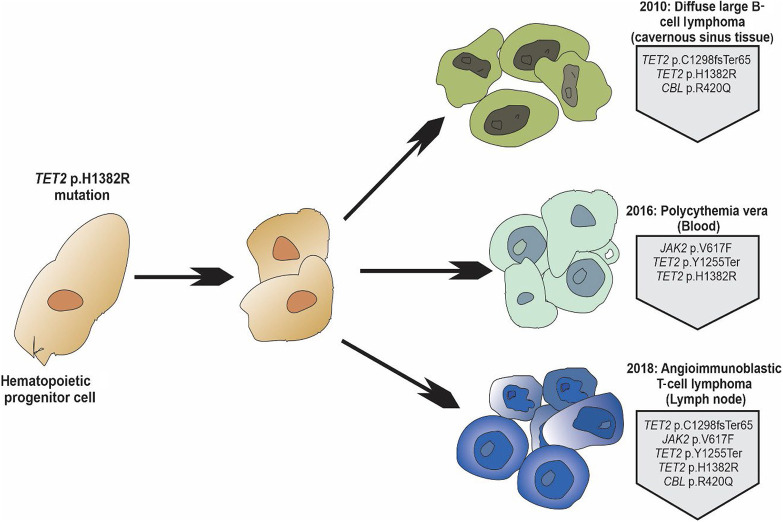
Schematic diagram illustrating the development of angioimmunoblastic T-cell lymphoma (AITL), polycythemia vera (PV), and diffuse large B-cell lymphoma (DLBCL) from a common hematopoietic progenitor precursor. The different neoplastic cells from the three different malignancies from different locations share the *TET2* and *CBL* mutations, supporting the idea of a common mutated progenitor cell.

We retrospectively confirmed and expanded the NGS analyses on all three tumor samples obtained from different anatomic sites at different times in the patient's course to evaluate the possibility of clonal relatedness via the identification of common shared mutations (Table 1). NGS was performed on two different platforms: the Illumina TruSight myeloid sequencing panel (34 genes) and the ArcherDx VariantPlex core myeloid panel (37 genes). The DLBCL sample showed three distinct mutations: (1) *TET2* p.Cys1298LeufsTer65 (c.3893delG), VAF = 18%; (2) *TET2* p.H1382R (c.4145A > G), VAF = 35%; and (3) *CBL* p.R420Q (c.1259G > A), VAF = 10% (Table 1; Fig. 3). The PV peripheral blood sample revealed four mutations: (1) *JAK2* p.Val617Phe (c.1849G > T), VAF = 80%; (2) *TET2* p.Tyr1255Ter (c.3765delC), VAF = 50%; (3) *TET2* p.H1382R (c.4145A > G), VAF = 53%; and (4) *CBL* p.R420Q (c.1259G > A), VAF = 2% (Table 1; Fig. 3). Last, the AITL sample demonstrated the following five mutations, each at comparably high VAFs across both platforms: (1) *JAK2* p.Val617Phe (c.1849G > T), VAF = 15%; (2) *TET2* p.Tyr1255Ter (c.3765delC), VAF = 12%–13%; (3) *TET2* p.Cys1298LeufsTer65 (c.3893delG), VAF = 18%–21%; (4) *TET2* p.H1382R (c.4145A > G), VAF = 37%; (5) and *CBL* p.R420Q (c.1259G > A), VAF = 25%–26% (Table 1; Fig. 3). The low VAF of *CBL* R420Q mutation in the peripheral blood sample was confirmed by Sanger sequencing. The summary of all the variants detected can be seen in Table 2. Overall, we identified up to five genomic alterations shared across multiple specimens, with a single prominent founding mutation co-occurring in all three tumor types.

**Table 2.** Summary of all detected variants

Gene	Chromosome	HGVS DNA reference	HGVS protein reference	Variant type	Predicted effect (substitution, deletion, etc.)	dbSNP/dbVar ID
*JAK2*	Chr 9	NM_004972.4:c.1849G > T	NP_004963.1:p.Val617Phe	SNV	Missense	rs77375493
*TET2*	Chr 4	NM_001127208.3:c.3765C > A	NP_001120680.1:p.Tyr1255Ter	SNV	Nonsense	n.i.
*TET2*	Chr 4	NM_001127208.3:c.3893del	NP_001120680.1:p.Cys1298LeufsTer65	Deletion	Frameshift	n.i.
*TET2*	Chr 4	NM_001127208.3:c.4145A > G	NP_001120680.1:p.His1382Arg	SNV	Missense	rs1131691943
*CBL*	Chr 11	NM_005188.4:c.1259G > A	NP_005179.2:p.Arg420Gln	SNV	Missense	rs267606708

(SNV) Single-nucleotide variant, (n.i.) not indicated.

## DISCUSSION

This case demonstrates a patient with three clinically distinct hematologic neoplasms that apparently arose as the result of differentiation from a common hematopoietic precursor (Fig. 3). The high VAF of a single mutation in particular, *TET2* H1382R (c.4145A > G), occurring in high proportion (37%–53%) of all sequencing reads from all three specimens mitigates against concerns of low-level peripheral blood contamination from clonal hematopoiesis of indeterminate potential (CHIP). *TET*2 mutations appear particularly promiscuous, as they have been shown to be associated with CHIP in healthy older adults; myeloid malignancies including acute myeloid leukemia (AML), myeloproliferative neoplasms (MPNs), and myelodysplastic/myeloproliferative neoplasms (MDS/MPNs); and a variety of mature B-cell and T-cell non-Hodgkin lymphomas. Previous case reports have demonstrated AITL following either a myeloid or lymphoid neoplasm derived from a common *TET2/DNMT3A* mutated stem cell population (Tiacci et al. 2018; Naganuma et al. 2020; Cheng et al. 2021; Attygalle et al. 2022; Yoshihara et al. 2022). Single-cell isolation techniques have confirmed that B cells or myeloid cells from AITL patients share *TET2* mutations (Couronné et al. 2012; Schwartz et al. 2017; Lewis et al. 2020). Our case is distinct in that it shows the development of B-cell lymphoma and chronic MPN harboring *TET2* mutations before the diagnosis of AITL. Cheng et al. (2021) reported one patient with *TET2* mutations who developed metachronous *JAK2*-positive PV followed by AITL. In such examples, no studies have been done showing what determines which type of malignancy will develop first. Indeed, the available data in the current case do not provide for an established “order” of clonal relatedness following the apparent founder effect exhibited by the mutation in *TET2* p.H1382R. The range of VAFs for each secondary mutation, along with the likelihood of admixed clonal leukocyte populations being present in bulk tumor analysis, prevents any definitive conclusions. We speculate that multiple intermediate steps, including clonal branches not represented here, were likely contributory to the development of each clinically apparent neoplasm.

AITL is one of the most common subtypes of peripheral T-cell lymphoma and is the prototype of the follicular helper T cell phenotype. AITL has been shown to be characterized by unique genomic alterations including *TET2*, *DNMT3A*, *IDH2*, and *RHOA* (Couronné et al. 2012; Sakata-Yanagimoto et al. 2014; Butzmann et al. 2020). *TET2* and *DNMT3A* mutations have also been associated with clonal hematopoiesis (CH) in healthy older adults (Xie et al. 2014). The CH-related genomic mutations have been identified in bone marrow myeloid-derived cells of AITL patients, supporting the evidence of a common mutated hematopoietic stem cell clone (Cheng et al. 2021). Furthermore, *TET2* mutations present in AITL have also been found in isolated CD34-positive cells, CD8-positive T cells, benign B cells, and tumor cells, supporting the notion of a mutated stem cell progenitor cell (Couronné et al. 2012; Schwartz et al. 2017; Cheng et al. 2021). However, *TET2* or *DNMT3A* mutations alone have been shown to be insufficient for tumor formation, and additional mutations are required to further drive the clonal expansion. Cheng et al. (2021) reported to have found additional mutations in 70% of their AITL cases. Expression of *RHOA*^G17V^ or *TET2* mutations alone in AITL mouse models does not lead to tumor formation unless the two genetic alterations are combined (Ng et al. 2018). In our patient, each malignant specimen type demonstrated a second *TET2* abnormality (presumably *trans* mutation[s] resulting in biallelic dysfunction), whereas the high-frequency *JAK2* p.V617F mutation (80% VAF) of the peripheral blood was associated with predominantly myeloid cellularity and a phenotypic MPN. Notably, patients with two or more pathogenic *TET2* genetic mutations with a VAF of ≥15% in the peripheral blood have been shown to have a high-risk factor (*P* = 0.0034, hazard ratio = 10.81) for the development of myeloid and B-cell malignancies (Cheng et al. 2021). In agreement with this, aging mice with *TET2* mutations have been demonstrated to have different hematolymphoid malignancies (Pan et al. 2017). Furthermore, *TET2* mutations in AITL have been shown to be associated with higher tumor burden, shorter progression-free survival, and advanced-stage disease (Lemonnier et al. 2012; Ye et al. 2021).

Interestingly, we also detected *CBL* p.R420Q mutations in all three samples. *CBL* is a member of a family of RING finger ubiquitin E3 ligases that regulate the signaling of tyrosine kinases (Schmidt and Dikic 2005; Tsygankov 2008). *CBL* mutations have been identified in different myeloid neoplasms including MDS/MPN and acute leukemia but are rarely found in lymphoid malignancies (Caligiuri et al. 2007; Sargin et al. 2007; McKeller et al. 2009; Reindl et al. 2009). *CBL* p.R420Q is a point mutation that inhibits the ubiquitin ligase functionality that is responsible for tyrosine kinase signaling. The R420Q mutation was first described in AML and was shown to inhibit Flt3 internalization and ubiquitination, leading to ligand-independent signaling of Flt3, which is able to transform myeloid cells in vitro (Sargin et al. 2007). *CBL* mutations have been shown to be found at similar frequencies in *JAK2 V617-*positive and -negative MPNs (Aranaz et al. 2012). Most of the *CBL* mutations have shown to be located in the highly conserved Ring finger domain and the linker domain, and alterations in the gene result in dysregulation of the targets downstream and enhanced cell proliferation rates. In this patient, the low VAF for the *CBL* p.R420Q mutation as detected in the peripheral blood makes it unlikely to be a founding genetic event but more likely to be committed to lymphoid cells present in the sample.

This report is limited by necessary reliance on bulk tissue NGS analysis. The possibility of “contamination” by nonmalignant CH cells in each specimen type must be considered. However, the relatively high VAF for each (with the exception of the *CBL* p.R420Q mutation in the peripheral blood) points toward a role for each mutation in the primary tumor cell type. Prior studies using single-cell sorting of neoplastic T cells and myeloid cells have demonstrated common shared *TET2* mutations as well as different mutations in each tumor, confirming clonal relatedness (Schwartz et al. 2017; Lewis et al. 2020). One further limitation here is the inability of routine NGS on tumor tissue to distinguish acquired somatic from germline mutations. The formal possibility exists that the common *TET2* p.H1382R mutation in this case could be a germline variant (sequencing of paired normal tissue would be required to exclude), but the deviation from 50% VAF for this finding mitigates against such concerns.

In summary, this case illustrates the clinical presentation of up to three distinct hematologic neoplasms in a single patient potentially rising from a common mutated hematopoietic precursor with substantial multipotency, populating erythroid/myeloid, and B and T lymphoid lineages (Fig. 3). This case provides an opportunity to study discrete hematolymphoid neoplasms occurring at different times and in disparate locations. This case raises interesting questions regarding the early detection and intervention of hematolymphoid neoplasms, especially in the context of the ongoing evolution of CH.

## METHODS

### Clinical Specimens

The patient was monitored and/or treated first at an outside institution and then at the University of Rochester Medical Center (URMC). Different specimens’ biopsies were collected at various time points during treatment.

### Specimen Processing and Morphologic Assessment

The specimens were then processed using the automated tissue processors Leica ASP300S and Leica Peloris II (Leica Biosystems Division of Leica Microsystems, Inc.). Four-micrometer section tissue slides were cut from the processed paraffin blocks and stained with H&E using H&E automated stainers (Sakura Finetek USA, Inc.; Leica Biosystems, Division of Leica Microsystems, Inc.). Immunohistochemical stains were performed using the following different antibodies: CD20 (clone L26), CD10 (clone 56C6), CD4 (clone SP35), CD79a (clone JCB11), BCL6 (clone LN22), BCL2 (clone bcl2/100/D5), ki-67 (clone MIB-1), C-MYC (EP121), CD2 (clone AB75), CD3 (polyclonal), CD5 (clone 4C7), CD4, CD21 (clone 1F8), CD23 (clone 1B12), PD1 (clone nat105), PAX5 (BSA; clone DAK-PAX5), CD7 (clone CBC.37), CD15 (clone Carb-3), CD30 (clone Ber-H2), and ALK-1 (clone CD246). Morphologic assessment of the H&E-stained FNA biopsy specimens, as well as interpretation of immunohistochemical stains, was performed by a board-certified anatomic pathologist. Wright–Giemsa stain was performed on peripheral blood samples with a Midas III Stainer (Fisher Scientific, part of Thermo Fisher Scientific; Sysmex America, Inc.; Sigma-Aldrich, Inc.).

### Flow Cytometric Immunophenotyping

Immunophenotyping assays were performed by the URMC Clinical Flow Cytometry Laboratory for standard clinical care using a Beckman Coulter Navios flow cytometer, FDA-approved 10-color ClearLLab lyophilized immunophenotyping tubes, and Kaluza C analysis software (Beckman Coulter Life Sciences). Peripheral blood samples were processed using a stain/lyse/wash protocol. Cell concentrations were adjusted to 3 × 10^6^/mL to 20 × 10^6^/mL to ensure optimal antibody staining, and the cells were washed three times before acquisition. Viability was assessed using 7AAD and CD45. Following morphological review of peripheral slides, 10-color analyses were performed for the following surface and cytoplasmic antigens: κ, λ, CD10, CD5, CD200, CD34, CD38, CD20, CD19, CD45, TCRδ/γ, CD4, CD2, CD56, CD5, CD7, CD8, CD3, CD45, CD16, CD7, CD10, CD13, CD64, CD34, CD14, HLA-DR, CD11b, CD45, CD15, CD123, CD117, CD13, CD33, CD34, CD38, HLA-DR, CD19, CD45, 6AC1, cy-TdT, cy-79a, CD34, CD22, CD19, CD45, 6AC2, cy-MPO, CD117, CD34, CD1a, cy-CD3, and CD45. Cells were gated to exclude debris (forward scatter vs. side scatter and time of flight), to exclude cell doublets (forward scatter height vs. forward scatter width), and to isolate leukocyte populations (CD45 vs. side scatter). Primary analysis and quality control were performed by the flow cytometry supervisor. Final gating and reporting were performed by a board-certified hematopathologist.

### NGS Testing

DNA was extracted using the Qiagen DNeasy blood and tissue kit per the manufacturer's instructions (Qiagen). Sequencing libraries were prepared for sequencing on the Illumina TruSight myeloid sequencing panel or on the ArcherDx VariantPlex core myeloid panel per the manufacturers’ protocols. The enriched DNA libraries were sequenced on an Illumina MiSeq instrument (version 3 chemistry, 300-bp paired-end reads; Illumina, Inc.; TruSight myeloid panel). FASTQ files were processed through vendor-provided bioinformatics pipelines. Variant call files were filtered to remove subthreshold calls with <500× coverage and/or VAF less than the defined, validated thresholds ranging from 1% to 5%, depending on the type of mutation, as follows: 5% for single=nucleotide variants (SNVs), 1% for insertion–deletion mutations <3 bp, and 5% for insertion–deletion mutations ≥3 bp. Clinically relevant mutations from this VAF were annotated manually by a board-certified molecular genetic pathologist and reported. Known pathogenic mutations with significant VAFs were determined from the tumor sequencing alone and using established public databases such as Catalogue of Somatic Mutations in Cancer (COSMIC), cBioPortal, Clinical Interpretations of Variants in Cancer, and OncoKB. The different variants were classified using the four-tier classification based on their level of clinical significance in cancer diagnosis, prognosis, and/or therapeutics (Richards et al. 2015; Li et al. 2017; Horak et al. 2022). Sequenced regions (i.e., mutational hotspot regions, consisting of the indicated exons) of the clinically ordered gene set for the TruSight myeloid panel and ArcherDx VariantPlex core myeloid panel are in Supplemental Tables 1 and 2.

## ADDITIONAL INFORMATION

### Data Deposition and Access

Consent documentation did not allow for submission of full sequencing data (FASTQ, BAM/BAI, and VCF) to external data repositories. The interpreted variants were submitted to ClinVar (https://www.ncbi.nlm.nih.gov/clinvar/) and can be found under accession numbers SCV004175181–SCV004175185.

### Ethics Statement

The patient signed the institution-approved, standard consent for clinical diagnostic testing by NGS, including an opt in/out clause for the use of genetic and other diagnostic information for research purposes. This consent mechanism does not allow for the sharing of genetic and other diagnostic information beyond that clinically relevant and reported in the manuscript.

### Acknowledgments

We thank the histology and flow laboratory at the University of Rochester Medical Center for their assistance.

### Author Contributions

D.N., A.W.A., and A.G.E. reviewed and analyzed the data, created the figures, and wrote the manuscript with input from P.G.R. and J.W.F. All authors read the manuscript and approved its final version.

### Competing Interest Statement

The authors have declared no competing interest.

### Referees

Wenbin Xiao

Anonymous
